# Supplementary material for: Matrilineal phylogeny and habitat suitability of the endangered spotted pond turtle (Geoclemys hamiltonii; Testudines: Geoemydidae): a two-dimensional approach to forecasting future conservation consequences
Source: PeerJ. 2023 Sep 6;11:e15975. doi: 10.7717/peerj.15975 (PMC10492536; doi:10.7717/peerj.15975)
Supplement: Supplemental Information 1 [file peerj-11-15975-s001.pdf]

Table S1. List of acquired Testudines species mitogenomes from GenBank database for phylogenetic analysis along with their size and IUCN status.

Sl. No.	Species	Subfamily	IUCN status	Accession number	Size (bp)
1	*Geoclemys hamiltonii*	Batagurinae	Endangered	OP344485	16509
2	*Geoclemys hamiltonii*	Batagurinae	Endangered	ON243873	16505
3	*Batagur affinis*	Batagurinae	Critically Endangered	OQ409915	16625
4	*Batagur kachuga*	Batagurinae	Critically Endangered	MZ562559	16517
5	*Batagur trivittata*	Batagurinae	Critically Endangered	KX817298	16463
6	*Pangshura sylhetensis*	Batagurinae	Critically Endangered	MK580979	16568
7	*Pangshura tentoria*	Batagurinae	Least Concern	MH795989	16657
8	*Cuora amboinensis*	Geoemydinae	Endangered	FJ763736	16708
9	*Cuora aurocapitata*	Geoemydinae	Critically Endangered	AY874540	16890
10	*Cuora bourreti*	Geoemydinae	Critically Endangered	JN020145	16649
11	*Cuora flavomarginata*	Geoemydinae	Endangered	LC574977	16721
12	*Cuora galbinifrons*	Geoemydinae	Critically Endangered	EU809939	17244
13	*Cuora mccordi*	Geoemydinae	Critically Endangered	OM327796	16551
14	*Cuora mouhotii*	Geoemydinae	Endangered	DQ659152	16837
15	*Cuora pani*	Geoemydinae	Critically Endangered	GQ889364	16922
16	*Cuora picturata*	Geoemydinae	Critically Endangered	JF712890	16623
17	*Cuora trifasciata*	Geoemydinae	Critically Endangered	KF574821	16675
18	*Cuora yunnanensis*	Geoemydinae	Critically Endangered	MT334601	16023
19	*Cyclemys atripons*	Geoemydinae	Endangered	EF067858	16500
20	*Cyclemys dentata*	Geoemydinae	Near Threatened	JN582334	16475
21	*Cyclemys fusca*	Geoemydinae	Least Concern	JX218031	16491
22	*Cyclemys oldhamii*	Geoemydinae	Endangered	JN582335	16656

23	*Cyclemys pulchristriata*	Geoemydinae	Endangered	JQ266015	16527
24	*Cyclemys tcheponensis*	Geoemydinae	Endangered	JQ277464	16593
25	*Geoemyda japonica*	Geoemydinae	Endangered	LC574978	16820
26	*Geoemyda spengleri*	Geoemydinae	Endangered	MH748151	17448
27	*Heosemys annandalii*	Geoemydinae	Critically Endangered	JF742646	16604
28	*Heosemys depressa*	Geoemydinae	Critically Endangered	JQ266017	16773
29	*Heosemys grandis*	Geoemydinae	Critically Endangered	KX816868	16581
30	*Mauremys annamensis*	Geoemydinae	Critically Endangered	HM131942	16844
31	*Mauremys caspica*	Geoemydinae	Least Concern	KC692465	16741
32	*Mauremys japonica*	Geoemydinae	Near Threatened	GU938833	16443
33	*Mauremys leprosa*	Geoemydinae	Vulnerable	KP100055	17067
34	*Mauremys megalocephala*	Geoemydinae	Endangered	HM132059	16783
35	*Mauremys mutica*	Geoemydinae	Critically Endangered	KP100056	16775
36	*Mauremys nigricans*	Geoemydinae	Endangered	KT951839	16779
37	*Mauremys reevesii*	Geoemydinae	Endangered	AP019398	16784
38	*Mauremys rivulata*	Geoemydinae	Least Concern	KP100054	16766
39	*Mauremys sinensis*	Geoemydinae	Critically Endangered	FJ871126	16461
40	*Notochelys platynota*	Geoemydinae	Vulnerable	HQ853256	16981
41	*Sacalia bealei*	Geoemydinae	Endangered	MT372822	16564
42	*Sacalia quadriocellata*	Geoemydinae	Critically Endangered	MT372821	16433
43	*Rhinoclemmys punctularia*	Rhinoclemmydinae	Least Concern	JN999706	14154
44	*Manouria emys*	Family Testudinidae	Critically Endangered	NC_007693	16455
