# Supplementary material for: Matrilineal phylogeny and habitat suitability of the endangered spotted pond turtle (Geoclemys hamiltonii; Testudines: Geoemydidae): a two-dimensional approach to forecasting future conservation consequences
Source: PeerJ. 2023 Sep 6;11:e15975. doi: 10.7717/peerj.15975 (PMC10492536; doi:10.7717/peerj.15975)
Supplement: Supplemental Information 2 [file peerj-11-15975-s002.pdf]

Table S2. Locality information of *Geoclemys hamiltonii* acquired from previous literature.

Sl. No.	Country/Locations	References
1	Bangladesh, India (Assam, Bihar, Jammu, Meghalaya, Punjab, Rajasthan, Uttar Pradesh, West Bengal), Nepal, Pakistan	*TTWG, 2021*
2	Pakistan: Thatta, Sujawal and Badin districts of Sindh province. (Amerji Dhand, Barrage Mori, Buhri Lake, Chabo Mori, Deh Janghisar, Fuleli Guni Dhand, Guni Mori, Hadero Lake, Haleji Lake, Jaffar Ali Lake, Jhal Dhand, Jubho Lagoon, Kadhan Lagoon, Karo Dhand, Keenjhar Lake, Laakhi Dhand, Mehboob Shah Dhand, Othko Dhand, Phoosna Lake, Rajo Khanani Dhand, Tando bago, Thari Dhand)	*Amtyaz et al., 2021*
3	India: Assam and Tripura (Chikan ata Than, Bokaghat; Gorokhiya Gohain Than, Sarbog; Haigrib Madhab, Hajo; Mata Chandika Devi Mandir, Guwahati; Nagshankar Mandir, Biswanath; Siva Saga, Siba Sagar; Ugrotara, Guwahati; Chandan Pukur mandir, Agartala)	*Barhadiya et al., 2020*
4	India: Delhi, South West District: Najafgarh Drain, Chawwla (28.56639°N 77.02777°E)	*Barhadiya & Ghosh, 2020*
5	Pakistan: Daphar Forest Sanctuary, Tropical thorn forests	*Saba et al., 2020*
6	India: Jammu and Kashmir State	*Sahi & Koul, 2020*
7	India: Assam (Dibrugarh and Tinsukia districts)	*Sengupta, Borah & Phukon, 2019*
8	Pakistan: Keenjhar Lake, Haleji Lake, Hadero Lake, Mehboob shah dhand, Karo dhand, Jabho lagoon, Jhal dhand, Amerji dhand, Theri dhand, Thari dhand, Ohtko dhand, Jaffarali Lake, Phoosna Lake, Buhri Lake, Kadhan lagoon, Rajo khanani Dhand, Manchar Lake, Badram dhand, Sanjri dhand, Bakriwalo Lake, Vero Lake, Wadisim Lake, Chotiari wetlands, Kharoro Dhand, Bakar Lake	*Khan, Kanwal & Ghalib, 2018*
9	Pakistan: Kasur, Rawalpindi, Islamabad, Chakwal. River Indus and Korang, rivers of Punjab, Hub Dam in Sindh and Baluchistan provinces, Khyber Pakhtun Khwa Province, Charsadda, Peshawar, Nowshera, Dera Ismail Khan, Sindh Province, Thatta, Badin, Sanghar and Sukkur	*Waqas et al., 2018*
10	India: Assam (Guwahati)	*Purkayastha, 2018*
11	India: Assam (Kaziranga National Park)	*Basumatary & Sharma, 2013*

12	India: Assam (Garakhia Gohair Than, Sarbhog; Madhab Mandir, Hajo; Kamakhya Temple Pond, Guwahati; Nagshankar Temple Pond, Biswanath Chariali; Athkhelia Namghar, Golaghat), Tripura (Rajbari pond, Agartala)	*Purkayastha et al., 2013*
13	Pakistan: Cholistan Desert	*Baig, Masroor & Arshad, 2008*
14	India: Gujarat	*Vyas, 2007*
15	India: South and North Gujarat	*Sharma, 2000*

Cross References:

Amtyaz S, Khan MZ, Kanwal R, Karl H-V. 2021. Population Status, Threats and Conservation of the Spotted Pond Turtle; *Geoclemys Hamiltonii* (Gray, 1830) (Geoemydidae) of Pakistan. *Journal of Zoological Research* **3**: 29-37.

Baig KJ, Masroor R, Arshad M. 2008. Biodiversity and ecology of the herpetofauna of Cholistan Desert, Pakistan. *Russian Journal of Herpetology* **15**: 193-205.

Barhadiya G, Ghosh C. 2020. Geographic Distribution: *Geoclemys hamiltonii* (Spotted Pond Turtle). India: Delhi: South West District. *Herpetological Review* **51**: 535.

Barhadiya G, Singh S, Ghosh C, Basumatary R, Purkayastha J. 2020. Diversity and conservation potential of captive chelonian colonies at temple ponds in north-east India. *Herpetological Bulletin* **154**, 12-17.

Basumatary R, Sharma DK. 2013. The turtle fauna of Kaziranga National Park, Assam, India with notes on natural history and conservation status. *Herpetology Notes* **6**: 59-72.

Khan MZ, Kanwal R, Ghalib SA. 2018. Study of freshwater turtles in selected districts of Sindh-Pakistan: threats, illegal trade and conservation. *Journal of Animal and Plant Sciences* **28**: 915-926.

Purkayastha J, Hassan AM, Islam H, Das J, Sarma M, Basumatary M, Sarma N, Chatterjee N, Singha S, Nair V, et al. 2013. Turtles of the Temple Pond of Kamakhya, Assam, India. *Reptile Rap* **15**: 11-15.

Purkayastha J. 2018. Urban biodiversity: an insight into the terrestrial vertebrate diversity of Guwahati, India. *Journal of Threatened Taxa* **10**: 12299-12316.

Saba A, Ijaz S, Aslam H, Kanwal R, Afsheen S. 2020. Diversity of amphibians and reptiles in Daphar Forest Sanctuary, district Mandi Bahauddin, Pakistan. *Journal of Wildlife and Ecology* **4**: 15-26.

Sahi DN, Koul S. 2020. *Annotated List of Amphibians and Reptiles of Jammu and Kashmir State*. In: Dar, G, Khuroo, A. (eds) Biodiversity of the Himalaya: Jammu and Kashmir State. Topics in Biodiversity and Conservation, vol 18. Springer, Singapore.

Sengupta D, Borah CG, Phukon J. 2019. Assessment of the Reptilian Fauna in the Brahmaputra Plains of Two Districts in Assam, India. *Amphibian and Reptile Conservation* **26**, 65-67.

Sharma RC. 2000. *Reptilia. In: Fauna of Gujarat (Part 1). Vertebrates*, p. 243-298. Alfred JRB Ed., Kolkata, India, Zoological Survey of India.

Vyas R. 2007. *Present conservation scenario of reptile fauna in Gujarat State, India*. Indian Forester. 1381-1394.

Waqas A, Javid A, Hussain A, Bukhari SM. 2018. Diversity and conservation of freshwater turtles in Pakistan: a review. *Biodiversity* **19**: 62-71.
