# Supplementary material for: Matrilineal phylogeny and habitat suitability of the endangered spotted pond turtle (Geoclemys hamiltonii; Testudines: Geoemydidae): a two-dimensional approach to forecasting future conservation consequences
Source: PeerJ. 2023 Sep 6;11:e15975. doi: 10.7717/peerj.15975 (PMC10492536; doi:10.7717/peerj.15975)
Supplement: Supplemental Information 3 [file peerj-11-15975-s003.pdf]

Table S3: Primary environmental and topographical variables used for ensemble modelling.

Sl. No.	Code	Variables description
**Bioclimatic**		
1.	bio_1	Annual Mean Temperature
2.	bio_2	Mean Diurnal Range (Mean of monthly (max temp - min temp)
3.	bio_3	Isothermality (BIO2/BIO7) (* 100)
4.	bio_4	Temperature Seasonality (standard deviation *100)
5.	bio_5	Max Temperature of Warmest Month
6.	bio_6	Min Temperature of Coldest Month
7.	bio_7	Temperature Annual Range (BIO5-BIO6)
8.	bio_8	Mean Temperature of Wettest Quarter
9.	bio_9	Mean Temperature of Driest Quarter
10.	bio_10	Mean Temperature of Warmest Quarter
11.	bio_11	Mean Temperature of Coldest Quarter
12.	bio_12	Annual Precipitation
13.	bio_13	Precipitation of Wettest Month
14.	bio_14	Precipitation of Driest Month
15.	bio_15	Precipitation Seasonality (Coefficient of Variation)
16.	bio_16	Precipitation of Wettest Quarter
17.	bio_17	Precipitation of Driest Quarter
18.	bio_18	Precipitation of Warmest Quarter
19.	bio_19	Precipitation of Coldest Quarter
**Topographic**		
20.	elevation_2	Elevation
21.	slope	Slope
**Anthropogenic**		
22.	hii	Human Influence Index
**Habitat**		
23.	distance_water	Euclidian distance from major water body
24.	water	Occurrence Intensity
25.	LULC (Categorical)	• Herbaceous Vegetation<br>• Permanent Water Bodies<br>• Herbaceous Wetland<br>• Cropland<br>• Bare/Sparse vegetation<br>• Shrubland<br>• Moss and Lichens<br>• Forest<br>• Buildup areas
