# Supplementary material for: Matrilineal phylogeny and habitat suitability of the endangered spotted pond turtle (Geoclemys hamiltonii; Testudines: Geoemydidae): a two-dimensional approach to forecasting future conservation consequences
Source: PeerJ. 2023 Sep 6;11:e15975. doi: 10.7717/peerj.15975 (PMC10492536; doi:10.7717/peerj.15975)

Figure S1. Maximum Likelihood (ML) phylogenetic tree based on the concatenated nucleotide sequences of 13 PCGs showing the phylogenetic position of *Geoclemys hamiltonii* and other Geoemydidae species.

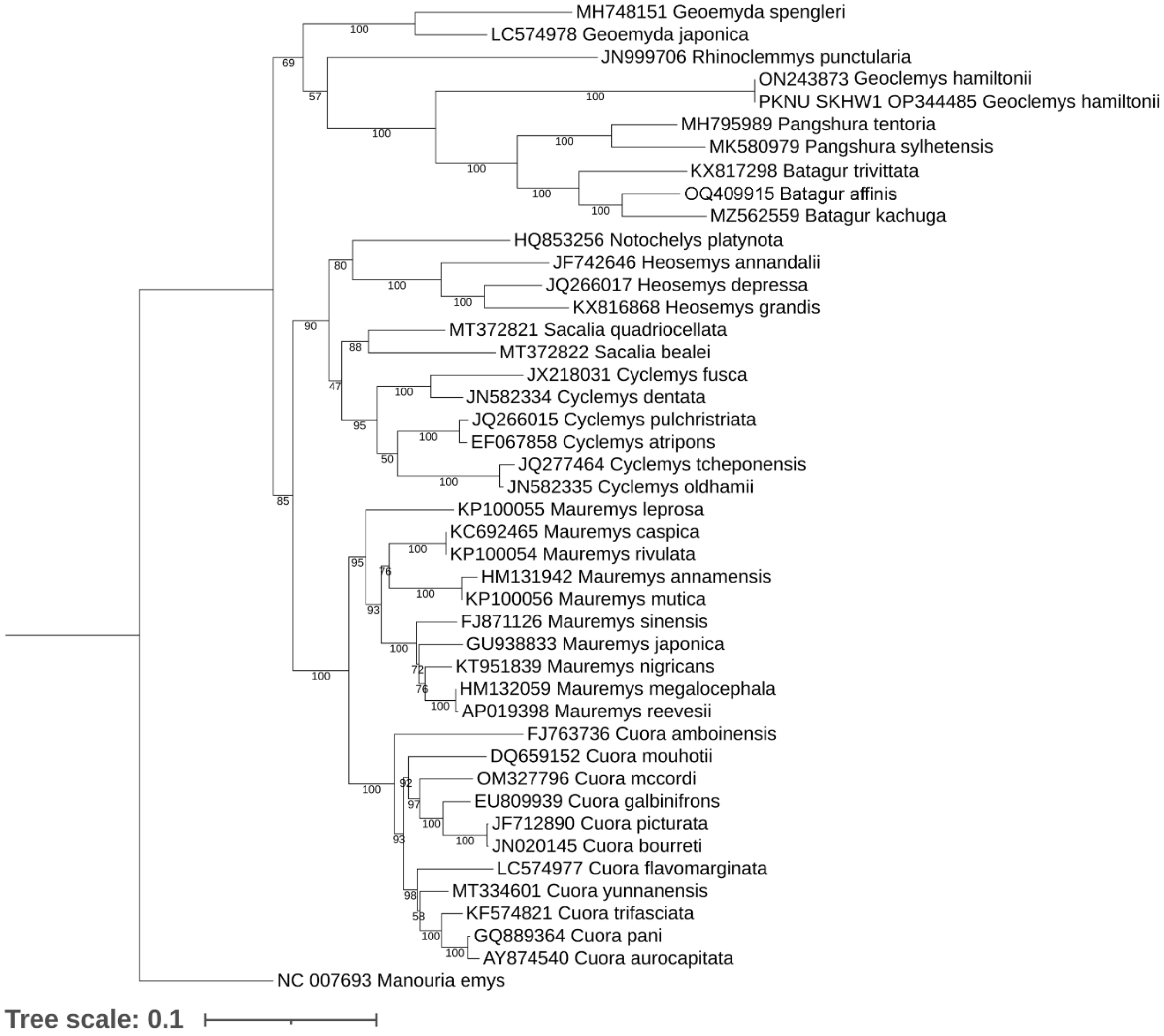

Supplement: Supplemental Information 4 [file peerj-11-15975-s004.pdf]
