# Supplementary material for: Matrilineal phylogeny and habitat suitability of the endangered spotted pond turtle (Geoclemys hamiltonii; Testudines: Geoemydidae): a two-dimensional approach to forecasting future conservation consequences
Source: PeerJ. 2023 Sep 6;11:e15975. doi: 10.7717/peerj.15975 (PMC10492536; doi:10.7717/peerj.15975)

Figure S2. Representing the final set of variables maps used for the distribution modelling of *Geoclemys hamiltonii*.

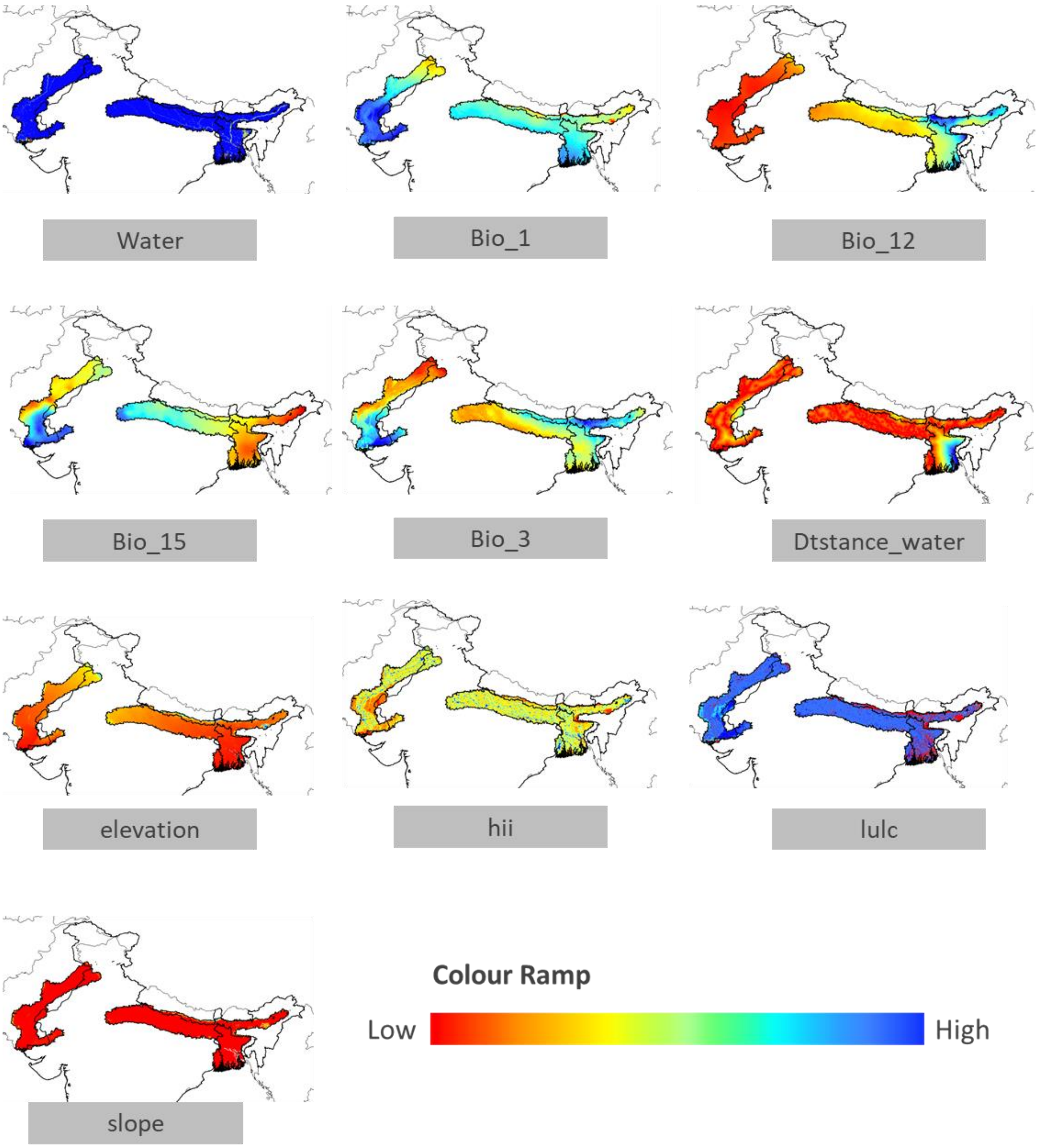

Supplement: Supplemental Information 5 [file peerj-11-15975-s005.pdf]
