# Supplementary material for: Matrilineal phylogeny and habitat suitability of the endangered spotted pond turtle (Geoclemys hamiltonii; Testudines: Geoemydidae): a two-dimensional approach to forecasting future conservation consequences
Source: PeerJ. 2023 Sep 6;11:e15975. doi: 10.7717/peerj.15975 (PMC10492536; doi:10.7717/peerj.15975)

Figure S3. Showing the response curves of the all the final selected variable (n=50) iteration. Curves show how each environmental variable affects the Maxent prediction. The curves show how the predicted probability of presence changes as each environmental variable is varied, keeping all other environmental variables at their average sample value. The curves show the mean response of the 50 replicate Maxent runs (red) and and the mean +/- one standard deviation (blue, two shades for categorical variables).

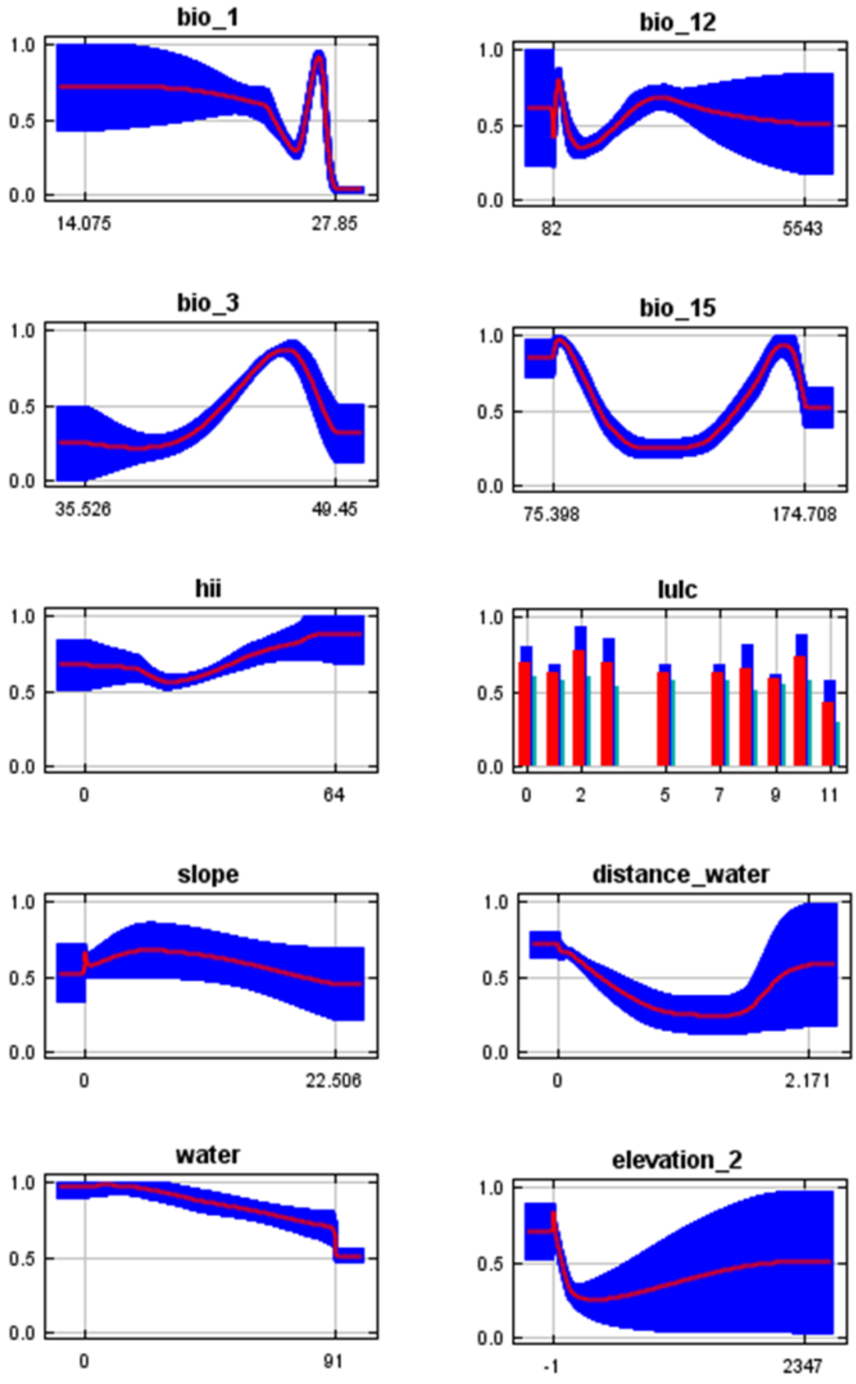

Supplement: Supplemental Information 6 — Curves show how each environmental variable affects the Maxent prediction. The curves show how the predicted probability of presence changes as each environmental variable is varied, keeping all other environmental variables at their average sample value. The curves show the mean response of the 50 replicate Maxent runs (red) and and the mean +/− one standard deviation (blue, two shades for categorical variables). [file peerj-11-15975-s006.pdf]
