# Supplementary material for: Matrilineal phylogeny and habitat suitability of the endangered spotted pond turtle (Geoclemys hamiltonii; Testudines: Geoemydidae): a two-dimensional approach to forecasting future conservation consequences
Source: PeerJ. 2023 Sep 6;11:e15975. doi: 10.7717/peerj.15975 (PMC10492536; doi:10.7717/peerj.15975)

Figure S4. Representing the training omission rate and predicted area as a function of the cumulative threshold, averaged over the replicate runs.

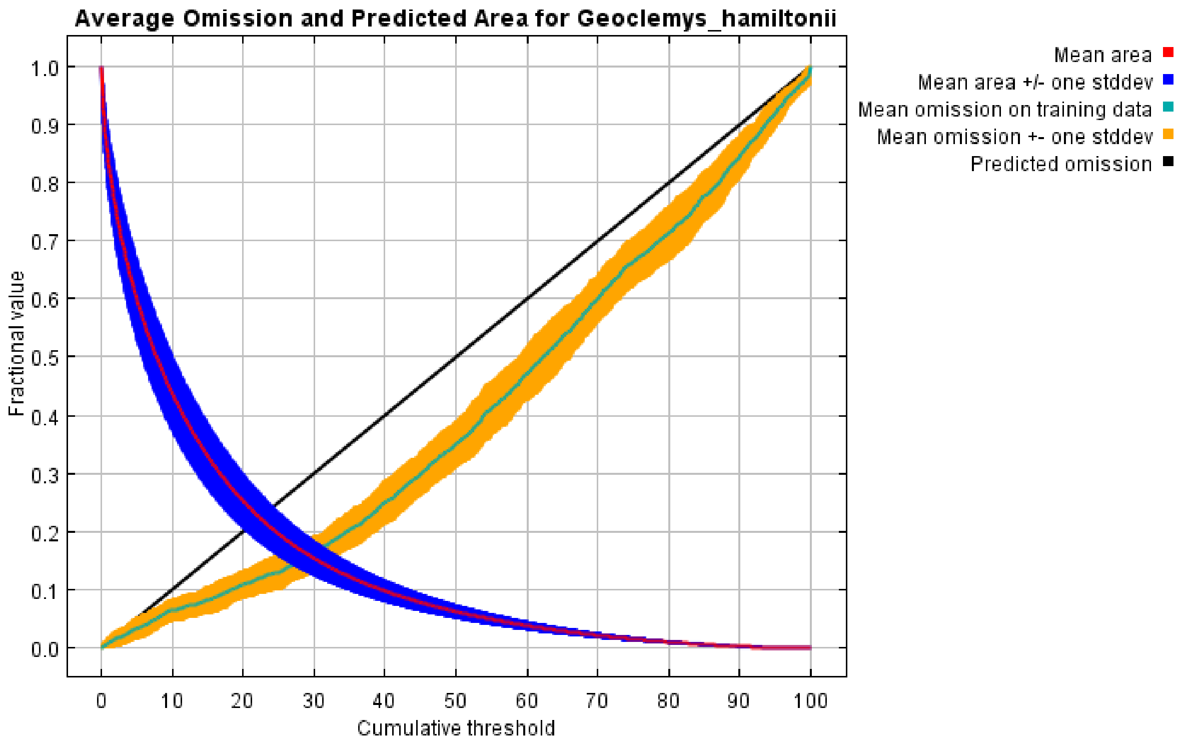

Supplement: Supplemental Information 7 [file peerj-11-15975-s007.pdf]
